# Supplementary material for: A Likelihood Approach for Real-Time Calibration of Stochastic Compartmental Epidemic Models
Source: PLoS Comput Biol. 2017 Jan 17;13(1):e1005257. doi: 10.1371/journal.pcbi.1005257 (PMC5240920; doi:10.1371/journal.pcbi.1005257)
Supplement: S1 File — (TAR.GZ) [file pcbi.1005257.s014.tar.gz › HSPH_Online-SI-Revision/PF08/n100-extreme/n100-extreme_PF_table8.pdf]

1	R0	{2.76052, 16.3913}	{2.17805, 16.5759}	{{2.17209, 16.5148}, {2.18329, 16.6192}}	{21.1002, 1.12647}	inf	3200.	6444.56	{6395.78, 6503.08}	101.393	0.304027	newly	1155.	2416.74	{2365., 2468.}	109.242	0.320584	Reff	0.245824	0.43079	{0.428722, 0.433504}	75.2429	ar	92 090.	87 674.6	{87 494., 87 848.}	4.79465
2	R0	{2.18202, 15.1734}	{2.20101, 20.}	{{2.19285, 20.}, {2.20653, 20.}}	{0.870387, 31.8096}	inf	3327.	7136.78	{7108.16, 7169.19}	114.511	0.331447	newly	1337.	2257.43	{2201., 2318.}	68.8429	0.227407	Reff	0.385257	0.409826	{0.407476, 0.411945}	6.37715	ar	84 264.	95 100.5	{94 446., 95 948.}	12.8601
3	R0	{2.57891, 12.5971}	{2.95726, 20.}	{{2.93372, 20.}, {2.97299, 20.}}	{14.6709, 58.7667}	inf	1362.	5807.67	{5778.29, 5837.54}	326.408	0.629821	newly	609.	1775.14	{1729., 1818.}	191.484	0.464535	Reff	0.262765	0.218337	{0.215979, 0.221489}	16.9081	ar	90 302.	98 585.3	{98 404., 98 805.}	9.17293
4	R0	{2.5856, 9.28508}	{3., 20.}	{{3., 20.}, {3., 20.}}	{16.0272, 115.399}	inf	433.	8026.68	{8007.36, 8044.02}	1753.74	1.26805	newly	254.	2453.26	{2386., 2509.}	865.85	0.98484	Reff	0.254526	0.214522	{0.213009, 0.215936}	15.7172	ar	90 312.	103 312.	{102 906., 103 803.}	14.3948
5	R0	{2.4406, 12.1341}	{2.85533, 20.}	{{2.84814, 20.}, {2.86211, 20.}}	{16.993, 64.8248}	inf	1891.	7835.09	{7819.56, 7852.52}	314.336	0.61735	newly	894.	2413.57	{2359., 2465.}	169.974	0.431254	Reff	0.297534	0.256211	{0.254871, 0.257949}	13.8885	ar	88 597.	97 693.2	{97 519., 97 959.}	10.267
6	R0	{2.72155, 15.4094}	{2.17879, 16.7367}	{{2.17536, 16.7099}, {2.18156, 16.7762}}	{19.9429, 8.6133}	inf	3044.	7268.16	{7234.16, 7308.}	138.77	0.377973	newly	1132.	2706.11	{2639., 2772.}	139.056	0.378428	Reff	0.252533	0.441693	{0.440533, 0.443326}	74.9053	ar	91 682.	88 808.9	{88 637., 88 942.}	3.13382
7	R0	{2.63781, 14.3935}	{2.13068, 20.}	{{2.12617, 20.}, {2.13385, 20.}}	{19.2254, 38.9516}	inf	2374.	11 160.7	{11 138.1, 11 180.6}	370.12	0.672209	newly	968.	3597.02	{3540., 3656.}	271.593	0.570028	Reff	0.252043	0.530768	{0.528653, 0.533366}	110.587	ar	91 192.	89 549.9	{89 286., 89 771.}	1.80074
8	R0	{2.82391, 16.104}	{2.14463, 16.6775}	{{2.14332, 16.6482}, {2.14665, 16.7213}}	{24.0545, 3.5612}	inf	3239.	7177.61	{7144.28, 7232.19}	121.6	0.345565	newly	1178.	2687.73	{2632., 2738.}	128.16	0.358183	Reff	0.232408	0.454082	{0.453137, 0.45473}	95.3816	ar	92 664.	87 317.	{87 161., 87 466.}	5.77029
9	R0	{2.80645, 14.6457}	{2.12847, 17.2951}	{{2.12717, 17.2767}, {2.12927, 17.3216}}	{24.1579, 18.0903}	inf	2875.	8877.32	{8853.42, 8913.13}	208.776	0.489642	newly	1160.	3233.52	{3169., 3292.}	178.752	0.445171	Reff	0.229399	0.488565	{0.487751, 0.489305}	112.976	ar	92 567.	88 468.1	{88 319., 88 633.}	4.42801
10	R0	{1.86213, 5.4271}	{3., 20.}	{{3., 20.}, {3., 20.}}	{61.1058, 268.521}	inf	95.	7244.39	{7231.57, 7257.57}	7525.67	1.88228	newly	92.	2209.6	{2162., 2255.}	2301.74	1.38046	Reff	0.460598	0.209249	{0.208159, 0.210229}	54.5702	ar	75 362.	102 890.	{102 644., 103 128.}	36.5271
11	R0	{2.27349, 14.6338}	{2.22645, 17.7392}	{{2.22154, 17.6562}, {2.23089, 17.8287}}	{2.06907, 21.221}	inf	2757.	5714.11	{5618.25, 5799.93}	107.258	0.316474	newly	1137.	2000.75	{1944., 2053.}	75.9675	0.245334	Reff	0.351982	0.390937	{0.388765, 0.393472}	11.0674	ar	85 805.	92 739.4	{92 518., 92 931.}	8.08155
12	R0	{2.77261, 14.1894}	{2.22181, 20.}	{{2.21524, 20.}, {2.22772, 20.}}	{19.8657, 40.9503}	inf	2393.	11 629.4	{11 606.5, 11 650.8}	385.976	0.686614	newly	959.	3731.33	{3664., 3807.}	289.086	0.590004	Reff	0.226855	0.499612	{0.497651, 0.501491}	120.234	ar	92 508.	91 395.6	{91 075., 91 718.}	1.20245
13	R0	{2.65402, 19.3935}	{2.19639, 20.}	{{2.19247, 20.}, {2.19934, 20.}}	{17.2428, 3.12734}	inf	6091.	9325.22	{9309.5, 9348.55}	53.0984	0.18497	newly	2008.	3001.76	{2953., 3060.}	49.49	0.174569	Reff	0.292659	0.521051	{0.519047, 0.524016}	78.0406	ar	91 184.	91 647.5	{91 369., 91 903.}	0.517953
14	R0	{1.75297, 4.45354}	{2.19173, 18.0255}	{{2.19173, 18.0255}, {2.19173, 18.0255}}	{25.0298, 304.745}	inf	33.	13 587.2	{13 587.2, 13 587.2}	41 073.2	2.61462	newly	37.	4844.48	{4770., 4916.}	12 993.2	2.11702	Reff	0.50468	0.565382	{0.565382, 0.565382}	12.0278	ar	71 263.	91 065.2	{90 857., 91 227.}	27.7875
15	R0	{1.99928, 7.1501}	{2.90676, 20.}	{{2.90295, 20.}, {2.90874, 20.}}	{45.3905, 179.716}	inf	273.	7378.39	{7369.38, 7393.39}	2602.71	1.4318	newly	197.	2261.63	{2215., 2310.}	1048.04	1.0599	Reff	0.411872	0.245961	{0.245452, 0.246741}	40.282	ar	79 602.	99 928.6	{99 804., 100 059.}	25.5353
16	R0	{2.67398, 10.3134}	{2.94296, 20.}	{{2.93919, 20.}, {2.94521, 20.}}	{10.059, 93.9225}	inf	624.	8264.67	{8255.25, 8273.99}	1224.47	1.12204	newly	342.	2535.09	{2476., 2586.}	641.254	0.869913	Reff	0.237957	0.239186	{0.238392, 0.240492}	0.516451	ar	91 289.	100 133.	{99 921., 100 336.}	9.68829
17	R0	{2.44634, 16.3226}	{2.21368, 20.}	{{2.21084, 20.}, {2.21647, 20.}}	{9.51039, 22.5295}	inf	3997.	8063.69	{8043.81, 8093.15}	101.744	0.304798	newly	1461.	2553.54	{2501., 2600.}	74.7803	0.242437	Reff	0.317486	0.420439	{0.418573, 0.422889}	32.4277	ar	88 710.	94 460.2	{94 144., 94 793.}	6.48205
18	R0	{2.02168, 10.1676}	{2.11178, 20.}	{{2.10868, 20.}, {2.11427, 20.}}	{4.4568, 96.7033}	inf	1489.	11 095.1	{11 081.1, 11 108.4}	645.141	0.872238	newly	847.	3639.51	{3569., 3709.}	329.694	0.633119	Reff	0.420772	0.635891	{0.634222, 0.637773}	51.1248	ar	80 299.	87 332.8	{87 080., 87 557.}	8.7595
19	R0	{2.89969, 15.009}	{2.23015, 20.}	{{2.22231, 20.}, {2.23401, 20.}}	{23.0902, 33.2534}	inf	2686.	11 781.4	{11 760., 11 809.6}	338.622	0.642089	newly	1026.	3776.46	{3697., 3843.}	268.076	0.565897	Reff	0.211938	0.497018	{0.494527, 0.500199}	134.511	ar	93 349.	91 777.8	{91 536., 92 044.}	1.68315
20	R0	{2.68933, 7.34928}	{3., 20.}	{{3., 20.}, {3., 20.}}	{11.5519, 172.136}	inf	136.	11 314.3	{11 300.2, 11 326.6}	8219.33	1.92009	newly	80.	3483.28	{3406., 3561.}	4254.1	1.63885	Reff	0.227975	0.254663	{0.253527, 0.255618}	11.7068	ar	91 574.	100 328.	{100 114., 100 554.}	9.55942
21	R0	{2.15352, 9.28074}	{2.94509, 20.}	{{2.94258, 20.}, {2.94711, 20.}}	{36.7572, 115.5}	inf	838.	7272.55	{7260.63, 7283.59}	767.846	0.938442	newly	504.	2230.26	{2178., 2283.}	342.512	0.64585	Reff	0.358733	0.238226	{0.237711, 0.239004}	33.5926	ar	83 845.	99 508.4	{99 382., 99 632.}	18.6814
22	R0	{2.21953, 9.04387}	{3., 20.}	{{3., 20.}, {3., 20.}}	{35.1638, 121.144}	inf	694.	6698.86	{6684.81, 6717.18}	865.254	0.984641	newly	414.	2042.22	{1997., 2095.}	393.29	0.693024	Reff	0.343938	0.201638	{0.2006, 0.2025}	41.3739	ar	84 856.	102 298.	{101 993., 102 571.}	20.555
23	R0	{2.21987, 14.5347}	{1.95169, 20.}	{{1.95025, 20.}, {1.9529, 20.}}	{12.0807, 37.6017}	inf	2885.	9970.2	{9961.32, 9982.86}	245.587	0.538558	newly	1192.	3255.78	{3196., 3311.}	173.136	0.436336	Reff	0.366057	0.610369	{0.609172, 0.611591}	66.7417	ar	85 067.	83 946.	{83 718., 84 201.}	1.31776
24	R0	{2.84268, 17.9659}	{1.95517, 20.}	{{1.95325, 20.}, {1.95624, 20.}}	{31.221, 11.322}	inf	4541.	10 368.5	{10 353.8, 10 380.2}	128.33	0.358563	newly	1477.	3392.91	{3345., 3445.}	129.716	0.361158	Reff	0.242139	0.626164	{0.624767, 0.627857}	158.596	ar	92 836.	83 802.8	{83 570., 84 072.}	9.73027
25	R0	{2.52706, 10.3679}	{2.10423, 20.}	{{2.09418, 20.}, {2.11006, 20.}}	{16.7321, 92.9031}	inf	746.	12 980.2	{12 952., 13 005.2}	1639.98	1.24054	newly	432.	4223.69	{4150., 4298.}	877.706	0.99017	Reff	0.269107	0.604002	{0.600664, 0.609757}	124.447	ar	89 623.	87 779.3	{87 505., 88 104.}	2.05718
26	R0	{2.62835, 19.1088}	{2.27878, 20.}	{{2.27366, 20.}, {2.2832, 20.}}	{13.2999, 4.66382}	inf	5501.	7484.62	{7457.59, 7511.24}	36.0593	0.133722	newly	1758.	2353.57	{2296., 2410.}	33.8777	0.126625	Reff	0.292378	0.387769	{0.385962, 0.390218}	32.6261	ar	90 908.	96 321.4	{96 040., 96 583.}	5.95477
27	R0	{2.76831, 19.4155}	{2.15657, 16.9394}	{{2.15138, 16.876}, {2.15855, 16.9847}}	{22.0978, 12.7534}	inf	4502.	5022.18	{4962.87, 5069.56}	11.5544	0.0474136	newly	1418.	1840.	{1792., 1892.}	29.7602	0.113053	Reff	0.252857	0.410479	{0.409096, 0.412259}	62.3361	ar	92 175.	89 418.7	{89 255., 89 554.}	2.9903
28	R0	{2.96609, 16.8903}	{3., 20.}	{{3., 20.}, {3., 20.}}	{1.14326, 18.4112}	inf	3132.	5467.2	{5413.26, 5528.99}	74.5595	0.24193	newly	1123.	1670.64	{1628., 1718.}	48.7658	0.172401	Reff	0.198639	0.202082	{0.201075, 0.203441}	1.7333	ar	94 024.	100 489.	{100 094., 100 824.}	6.87623
29	R0	{2.17046, 14.39}	{1.98183, 20.}	{{1.97766, 20.}, {1.98446, 20.}}	{8.69096, 38.9854}	inf	3337.	10 156.6	{10 142.3, 10 171.3}	204.363	0.483392	newly	1397.	3314.53	{3246., 3374.}	137.261	0.375183	Reff	0.390705	0.5984	{0.596516, 0.600392}	53.1593	ar	84 001.	85 425.	{85 167., 85 683.}	1.69521
30	R0	{2.88772, 12.4647}	{3., 20.}	{{3., 20.}, {3., 20.}}	{3.88819, 60.4531}	inf	1402.	8075.36	{8067.34, 8086.75}	475.988	0.760413	newly	651.	2468.2	{2415., 2527.}	279.14	0.57874	Reff	0.203498	0.220458	{0.219665, 0.221351}	8.33423	ar	93 273.	101 480.	{101 217., 101 717.}	8.79935
31	R0	{2.46831, 8.14308}	{3., 20.}	{{3., 20.}, {3., 20.}}	{21.5406, 145.607}	inf	300.	9531.35	{9514.06, 9547.63}	3077.12	1.50203	newly	189.	2910.96	{2844., 2967.}	1440.19	1.18752	Reff	0.280894	0.227817	{0.226501, 0.229133}	18.8957	ar	88 762.	103 227.	{102 838., 103 619.}	16.296
32	R0	{1.93236, 7.38987}	{3., 20.}	{{3., 20.}, {3., 20.}}	{55.2506, 170.641}	inf	389.	6490.41	{6479.02, 6500.53}	1568.49	1.22232	newly	288.	1983.72	{1935., 2024.}	588.792	0.838016	Reff	0.437467	0.209275	{0.20853, 0.210003}	52.162	ar	77 654.	100 952.	{100 803., 101 074.}	30.0023
33	R0	{2.82251, 17.6954}	{2.90299, 20.}	{{2.87071, 20.}, {2.93465, 20.}}	{2.85147, 13.0237}	inf	4866.	6679.04	{6528.75, 6867.02}	37.2594	0.137468	newly	1670.	2049.07	{1983., 2123.}	22.6988	0.0886734	Reff	0.244147	0.239212	{0.231175, 0.246302}	2.50006	ar	92 745.	98 480.6	{98 001., 98 829.}	6.18426
34	R0	{1.87083, 12.3994}	{2.25828, 20.}	{{2.24151, 20.}, {2.27003, 20.}}	{20.7103, 61.2981}	inf	3095.	7877.44	{7817.5, 7974.42}	154.521	0.405713	newly	1528.	2506.07	{2437., 2557.}	64.0098	0.214787	Reff	0.498595	0.458493	{0.452393, 0.467367}	8.043	ar	76 282.	94 440.6	{93 751., 95 114.}	23.8046
35	R0	{2.95779, 16.3003}	{2.17316, 16.2334}	{{2.1703, 16.216}, {2.17453, 16.2575}}	{26.5275, 0.410403}	inf	3818.	7754.16	{7727.71, 7789.88}	103.095	0.307697	newly	1428.	2977.	{2929., 3033.}	108.473	0.319008	Reff	0.209086	0.455401	{0.454455, 0.456324}	117.805	ar	93 873.	87 941.	{87 786., 88 086.}	6.31921
36	R0	{2.18718, 14.261}	{2.17848, 16.1705}	{{2.1693, 16.089}, {2.18513, 16.2742}}	{0.398776, 13.3898}	inf	3033.	4742.77	{4615.24, 4884.9}	56.3721	0.194045	newly	1295.	1806.1	{1746., 1869.}	39.4672	0.144336	Reff	0.381269	0.400007	{0.39688, 0.404476}	4.91456	ar	84 379.	88 498.1	{88 162., 88 815.}	4.88165
37	R0	{2.44912, 9.31118}	{3., 20.}	{{3., 20.}, {3., 20.}}	{22.493, 114.796}	inf	559.	7392.22	{7379.18, 7407.26}	1222.4	1.12136	newly	306.	2261.1	{2212., 2317.}	638.922	0.868522	Reff	0.289559	0.216424	{0.215384, 0.217432}	25.2575	ar	88 442.	102 089.	{101 776., 102 364.}	15.4304
38	R0	{2.05949, 5.72514}	{3., 20.}	{{3., 20.}, {3., 20.}}	{45.6671, 249.336}	inf	81.	10 156.9	{10 151.7, 10 163.}	12 439.4	2.09828	newly	76.	3120.04	{3054., 3186.}	4005.32	1.61329	Reff	0.380141	0.244152	{0.243528, 0.244824}	35.7734	ar	81 587.	100 648.	{100 378., 100 888.}	23.363
39	R0	{1.75505, 8.52404}	{2.34029, 20.}	{{2.32342, 20.}, {2.34934, 20.}}	{33.3462, 134.631}	inf	1029.	8496.19	{8466.4, 8527.58}	725.674	0.916807	newly	719.	2669.34	{2612., 2727.}	271.257	0.569613	Reff	0.527357	0.381254	{0.377395, 0.38694}	27.7049	ar	70 925.	97 344.2	{96 798., 97 858.}	37.2495
40	R0	{2.24724, 13.7861}	{2.54571, 18.3987}	{{2.32837, 17.3604}, {2.90132, 20.}}	{13.2814, 33.458}	inf	2420.	5000.99	{4798.42, 5254.54}	106.653	0.314996	newly	1045.	1682.9	{1456., 1847.}	61.0431	0.204988	Reff	0.360008	0.314265	{0.235854, 0.362557}	13.123	ar	85 257.	99 663.2	{97 214., 103 263.}	16.8974
41	R0	{1.77635, 8.62406}	{2.3441, 14.5696}	{{2.34235, 14.5036}, {2.35709, 14.6118}}	{31.9617, 68.9408}	inf	882.	3563.3	{3517.37, 3587.57}	304.003	0.606281	newly	608.	1482.84	{1442., 1528.}	143.888	0.387004	Reff	0.494927	0.399016	{0.394574, 0.402457}	19.3787	ar	72 941.	99 027.9	{98 706., 99 264.}	35.7644
42	R0	{1.80397, 5.4932}	{3., 20.}	{{3., 20.}, {3., 20.}}	{66.2999, 264.087}	inf	102.	7316.89	{7310.07, 7324.74}	7073.42	1.85573	newly	82.	2235.78	{2181., 2288.}	2626.56	1.43555	Reff	0.48442	0.212156	{0.211051, 0.212969}	56.2042	ar	73 232.	101 347.	{101 059., 101 644.}	38.3921
43	R0	{2.83096, 16.7077}	{1.90906, 20.}	{{1.90797, 20.}, {1.90952, 20.}}	{32.5648, 19.7053}	inf	3887.	11 979.8	{11 967.8, 11 983.2}	208.202	0.488834	newly	1377.	3983.92	{3907., 4046.}	189.319	0.461339	Reff	0.237546	0.729932	{0.72932, 0.730605}	207.28	ar	92 740.	82 191.6	{81 899., 82 447.}	11.3742
44	R0	{2.73678, 11.6021}	{3., 20.}	{{3., 20.}, {3., 20.}}	{9.61787, 72.3826}	inf	851.	6419.58	{6408.48, 6433.79}	654.357	0.877576	newly	393.	1961.16	{1909., 2017.}	399.023	0.698027	Reff	0.225237	0.209823	{0.208891, 0.210673}	6.84325	ar	92 003.	100 274.	{100 046., 100 498.}	8.98952
45	R0	{2.14235, 6.98601}	{2.94467, 20.}	{{2.94305, 20.}, {2.94601, 20.}}	{37.4505, 186.286}	inf	172.	8169.4	{8162.65, 8176.53}	4649.65	1.67666	newly	135.	2503.98	{2448., 2558.}	1754.8	1.26823	Reff	0.36707	0.239382	{0.238777, 0.240358}	34.7858	ar	82 974.	99 536.4	{99 381., 99 707.}	19.9609
46	R0	{2.23409, 9.21289}	{2.84924, 20.}	{{2.84293, 20.}, {2.85173, 20.}}	{27.5346, 117.087}	inf	866.	8821.45	{8805.41, 8846.29}	918.643	1.00802	newly	520.	2720.2	{2662., 2772.}	423.115	0.71854	Reff	0.341659	0.264397	{0.263443, 0.266733}	22.6139	ar	85 163.	99 316.2	{99 037., 99 510.}	16.619
47	R0	{2.12258, 8.15265}	{3., 20.}	{{3., 20.}, {3., 20.}}	{41.3374, 145.319}	inf	433.	7239.98	{7207.48, 7269.24}	1572.05	1.22325	newly	280.	2216.09	{2169., 2272.}	691.461	0.898356	Reff	0.375293	0.212145	{0.210835, 0.213415}	43.4722	ar	82 690.	104 518.	{103 977., 104 918.}	26.398
48	R0	{2.71771, 10.4636}	{3., 20.}	{{3., 20.}, {3., 20.}}	{10.3871, 91.1388}	inf	924.	8818.19	{8805.12, 8832.53}	854.35	0.979707	newly	498.	2699.71	{2632., 2754.}	442.11	0.734022	Reff	0.232147	0.21958	{0.21843, 0.220618}	5.41345	ar	91 716.	102 027.	{101 694., 102 289.}	11.2424
49	R0	{2.44369, 6.66477}	{3., 20.}	{{3., 20.}, {3., 20.}}	{22.7652, 200.085}	inf	49.	9747.95	{9740.16, 9756.18}	19 793.8	2.29872	newly	43.	2988.33	{2933., 3051.}	6849.6	1.84191	Reff	0.273693	0.231841	{0.23113, 0.232519}	15.2917	ar	88 827.	103 235.	{102 874., 103 517.}	16.22
50	R0	{2.77383, 17.9775}	{2.16312, 20.}	{{2.15948, 20.}, {2.16484, 20.}}	{22.0167, 11.2502}	inf	3843.	8570.12	{8557.89, 8584.37}	123.006	0.348316	newly	1236.	2720.4	{2664., 2762.}	120.097	0.342562	Reff	0.244319	0.455486	{0.454077, 0.457708}	86.4309	ar	92 334.	91 599.7	{91 205., 91 893.}	0.795254
